# Nano-Encapsulated Melatonin: A Promising Mucosal Adjuvant in Intranasal Immunization against Chronic Experimental *T. gondii* Infection

**DOI:** 10.3390/tropicalmed7120401

**Published:** 2022-11-27

**Authors:** Doaa E. Said, Eglal I. Amer, Eman Sheta, Shaimaa Makled, Hala E. Diab, Fadwa M. Arafa

**Affiliations:** 1 Department of Medical Parasitology, Faculty of Medicine, Alexandria University, Alexandria 5424041, Egypt; 2 Department of Pathology, Faculty of Medicine, Alexandria University, Alexandria 5424041, Egypt; 3 Department of Pharmaceutics, Faculty of Pharmacy, Alexandria University, Alexandria 21521, Egypt

**Keywords:** *Toxoplasma gondii*, melatonin, antioxidant, immunization, intranasal, Ig A, oxidative stress

## Abstract

Melatonin (MLT) is now emerging as one of the universally accepted immunostimulators with broad applications in medicine. It is a biological manipulator of the immune system, including mucosal ones. MLT was encapsulated in solid lipid nanoparticles (SLNs), then 100 mg/kg/dose of MLT-SLNs was used as an adjuvant of *Toxoplasma* lysate antigen (TLA). Experimental mice were intra-nasally inoculated with three doses of different regimens every two weeks, then challenged with 20 cysts of *T. gondii* Me49 strain, where they were sacrificed four weeks post-infection. Protective vaccine efficacy was evident via the significant brain cyst count reduction of 58.6%, together with remarkably high levels of humoral systemic and mucosal anti-*Toxoplasma* antibodies (Ig G, Ig A), supported by a reduced tachyzoites invasion of Vero cells in vitro upon incubation with sera obtained from these vaccinated mice. A cellular immune response was evident through the induction of significant levels of interferon-gamma (IFN γ), associated with morphological deteriorations of cysts harvested from the brains of vaccinated mice. Furthermore, the amelioration of infection-induced oxidative stress (OS) and histopathological changes were evident in mice immunized with TLA/MLT-SLNs. In conclusion, the present study highlighted the promising role of intranasal MLT-SLNs as a novel mucosal adjuvant candidate against chronic toxoplasmosis.

## 1. Introduction

Toxoplasmosis is a neglected tropical parasitic disease of poverty caused by the obligate intracellular apicomplexan protozoan, *Toxoplasma gondii.* It was recently described as malaria’s neglected cousin, besides being a leading cause of death attributed to foodborne illness [1,2]. Approximately one-third of the global human population is estimated to be infected, however, seroprevalences may reach up to 90% in highly endemic regions [1]. Unfortunately, the true magnitude of the disease is not known because toxoplasmosis is not a nationally reportable disease. Although asymptomatic infection with *T. gondii* is common in humans, there is an increased argument that the occult effects of latent *T. gondii* infection likely outweigh the recognized overt morbidity caused by toxoplasmosis [1]. Moreover, certain individuals including immunologically impaired patients and fetuses are at high risk for severe or life-threatening toxoplasmosis. Therefore, ignorance of toxoplasmosis could yield a public health disaster-in-waiting, unless control measures are urgently designed.

Vaccination has been considered an optimal approach for the prevention and control of toxoplasmosis [3]. Many factors are impacting the efficacy of a vaccine including vaccine type, formulation, adjuvant, dosing, and vaccine administration-related parameters [4]. Regarding vaccine type, many forms of recombinant proteins and/or DNA vaccines have been evaluated. Although they provided partial protection against toxoplasmosis, they were somehow stage-specific [5]. For that reason, a vaccine representing the different life stages of the parasite is likely to confer reasonable protection against *T. gondii* infection. Additionally, accumulating evidence points to multi-antigen immunizations are necessary for the development of an effective vaccine against complex pathogens, such as parasites. *Toxoplasma* lysate antigen (TLA) covers a wide range of potential protective antigens which consist mainly of sugars, lipids, and peptides [6]. The antigenicity of TLA is well established for the murine immune system for its ability to activate both innate and adaptive immune responses [5,7]. However, it was found that TLA is incapable of providing protection against toxoplasmosis when administrated alone [8].

The vaccine administration-related parameters were discussed in only a limited number of studies for their impact on vaccine efficiency. Indeed, the route of delivery can affect the vaccine localization which may power the priming of immune cells with substantial local and systemic immune responses [9]. Several problems have been associated with injected vaccines including safety, compliance, partial or no protection at mucosal sites, and the high cost of mass immunization, particularly in resource-poor developing countries. Therefore, the induction of protective immunity at both the mucosal and systemic levels by targeting mucosal compartments remains a major challenge [10]. Since *T. gondii* infection results from the ingestion of an oocyst, cyst, or pseudocyst, the intestinal mucosal barrier represents the primary defense against infection. Consequently, designing a vaccination approach that provokes a potent defense at the invasion site and maintains long-lasting protective immunity is a reasonable strategy [11]. The majority of mucosal vaccines are administered by the oral and nasal routes. However, the intranasal route requires fewer antigens than the oral route, owing to the presence of less proteolytic activity in the nasal cavity. Additionally, the intra-nasally administered antigen can penetrate the nasal epithelium, where it is processed by the antigen-presenting cells with activation of T and B cells that develop into IgA plasma cells and enter circulation via the thoracic duct, thus, reaching distant mucosal sites (e.g., intestines, respiratory tract, genital tract, and salivary glands) [11].

Therefore, mucosal vaccines, especially nasal ones, are a promising alternative to injected vaccines. However, the major limitations regarding the use of the nasal route are mostly related to the choice of suitable mucosal adjuvants and delivery systems whose potency needs to be carefully balanced with their potential toxicity [12]. Indeed, adjuvants are critical components in most clinical vaccines, owing to their ability to shape the quantity and quality of immune responses. Furthermore, their role in triggering and specifically directing the desired responses has been proven [13]. Many adjuvants have been previously evaluated in several studies [14,15]. However, most of them were never accepted for routine vaccination, owing to safety concerns such as acute toxicity and the possibility of delayed side effects [15]. Therefore, new efficient and safe adjuvants are strongly desired in designing prophylactic anti-*T. gondii* vaccines.

Considering the role of oxidative stress in the course of toxoplasmosis and the host immune defense, naturally occurring antioxidants with immune-modulatory action could be sensible candidates as vaccine adjuvants. Therefore, the scientific world is now in search of effective bio-modulators with antioxidant effects which can be used for the optimum modulation of the immune system [16].

Melatonin (MLT, N-acetyl-5-methoxytryptamine) is a ubiquitous molecule with natural and powerful antioxidant, anti-inflammatory, anti-apoptotic, and many other crucial properties. In particular, melatonin can diffuse and easily cross all physiological barriers, such as the placenta or the blood–brain barrier (BBB) due to its amphiphilic characteristics [17]. It can enter all cells of the body, influencing the function of a variety of tissues. Moreover, melatonin is not exclusively produced in the pineal gland, but it is also locally synthesized in several cells and tissues, such as the innate immune system, thus, suggesting its immunomodulatory role. Since the immunomodulatory effect of melatonin has been proven to enhance the magnitude and quality of immune responses specific to certain antigens, this has raised the possibility of using melatonin to design a novel vaccine adjuvant system [18].

Although MLT is easily absorbed from the mucosa, it has several obstacles to its use such as sensitivity to oxidation, high oral metabolism, and low oral bioavailability, thus, limiting its use in clinics. Hence, intranasal delivery provides an alternative to oral administration, particularly in unconscious patients or those with gastrointestinal problems [19]. Besides, the usually used melatonin solution tends to have low stability on storage and needs to be freshly prepared. These problems have led the researchers to focus on designing an efficient and proper delivery system alongside new routes of administration [20].

Nanotechnology has found its way into the fields of biology and medicine. Solid lipid nanoparticles (SLNs) have been evaluated in several trials with promising results that could be beneficial. They are constituted by lipids that remain solid at both the human body and room temperature—this is important when a controlled release is the main goal to be achieved. Interestingly, they possess several benefits such as higher adjuvant stability, incorporation of both hydrophilic and lipophilic substances, absence of toxicity, easy large-scale production, and the possibility of lyophilization [21].

Given that, the present work has been conducted to evaluate the potential effect of a naturally occurring antioxidant; MLT when loaded in SLNs is a promising mucosal adjuvant in improving the immunogenicity of TLA in the intranasal immunization against chronic murine toxoplasmosis.

## 2. Materials and Methods

### 2.1. Parasite

Two strains of *T. gondii* were used in the current study. They were maintained in the Medical Parasitology Department, Alexandria University Faculty of Medicine. The virulent RH HXGPRT (−) strain was used for antigen preparation, tachyzoite invasion, and replication assay. The avirulent ME49 strain was used for challenging the infection. Tachyzoites of the virulent *T. gondii* RH strain were maintained by a serial intra-peritoneal (IP) passage in laboratory-bred Swiss albino mice. Each naive Swiss albino mouse was inoculated intraperitoneally (IP) with 10^4^ tachyzoites. Then, the infected mice were sacrificed after five days by cervical dislocation [7]. Meanwhile, the cystogenic Me49 strain of *T. gondii* was maintained by a serial passage in Swiss albino mice every 2 months. Mice were orally inoculated by gavage with brain homogenate suspension containing *T. gondii* cysts (10 cysts/mouse). Eight weeks post-infection (PI), mice were sacrificed, brains were homogenized in a glass homogenizer (Wheaton, IL, USA) with normal saline, then brain suspensions from chronically infected mice were used for a subsequent experimental infection [22].

### 2.2. Antigen Preparation

Tachyzoites of the virulent *T. gondii* RH strain were obtained from the peritoneal fluid of infected mice and further processed for antigen preparation according to EL-Malky et al., 2014 [7]. Protein concentration was quantified by the Bradford method using a Bio-Rad protein assay reagent [23]. The TLA was aliquoted and stored at −20 °C until further use.

### 2.3. Preparation of Plain and MLT-SLNs

The preparation of plain and adjuvant-loaded SLNs was carried out in the department of Pharmaceutics, Alexandria Faculty of Pharmacy. A modified melt emulsification technique was used in the preparations. [24] The plain SLNs were prepared by heating the oil phase (OP) consisting of compritol 888 (Gattefossé, Saint-Priest, France) (0.6% *w*/*v*) at 85 °C (above the melting point of lipid). Then, the internal aqueous phase (AP) consisting of 4% PVA (Mowiol 4–88) (Mw ~31,000) (Kurary Specialties Europe GmbH, Frankfurt, Germany) at the same temperature was added to the mixture dropwise and homogenized using a high-shear homogenizer at 20,000× *g* rpm for 10 min to form a coarse pre-emulsion. The mixture was dispersed into cold water (the internal aqueous phase to external aqueous phase ratio is 3:2) which was maintained in an ice bath and homogenized by a high-shear homogenizer at 10,000× *g* rpm for 10 min. The formulation was subjected to centrifugation at 10,000× *g* rpm for 10 min and the supernatant was removed. The sediment was withdrawn and used for further characterization. To prepare melatonin-loaded SLNs, MLT (Sigma-Aldrich, St. Louis, MI, USA) (0.2% *w*/*v*) was added to the oil phase. The formulation variables (lipid and emulsifier types and concentrations in addition to a drug-to-lipid ratio) and formulation procedure (homogenization speed and time) were optimized for optimal colloidal characteristics and maximum adjuvant loading (data not shown). Lyophilization (Telestar LyoQuest Lyophilizer (Terrassa, Spain)) was carried out for SLN concentration and adjuvant-loading determination.

### 2.4. Characterization of the Prepared Nanoparticles

#### 2.4.1. Colloidal Characterization

Plain and MLT-loaded SLN dispersions were characterized in terms of particle size analysis, polydispersity index (PDI), and zeta potential using Malvern Zetasizer Nano ZS, Malvern Instruments, (Malvern, UK) in comparison to the freshly prepared plain SLNs [25]. Results were presented as the mean of 3 replicates.

#### 2.4.2. Morphological Characterization

Particles were examined under transmission electron microscopy TEM, model JEM-100CX (JEOL, Japan) for morphological characterization [25].

#### 2.4.3. Determination of Entrapment Efficiency and Adjuvant Loading

Entrapment efficiency (EE) was determined indirectly based on a modified centrifugal ultrafiltration technique using a Centrisart^®^-I tube (MWCO 300 kDa, Sartorius AG, Goettingen, Germany). Then, the adjuvant content in the ultrafiltrate was analyzed and quantified using a UV spectrophotometer at wavelength 278 nm (Figures S1 and S2 Supplementary Data) [25].

The *EE* was determined as follows:

$$EE\;\left(\%\right)=\left[\frac{\mathrm{Concentration}\;\mathrm{of}\;\mathrm{adjuvant}\;\mathrm{in}\;\mathrm{the}\;\mathrm{SLNs}\;\left(\mathrm{mg}/\mathrm{mL}\right)}{\mathrm{Initial}\;\mathrm{adjuvant}\;\mathrm{concentration}\;\left(\mathrm{mg}/\mathrm{mL}\right)}\right]\times 100$$

The adjuvant loading (*AL*) was also determined by quantifying the amount of adjuvant in a given weight of freeze-dried SLNs.

$$AL\;\left(\%\right)=\left[\frac{\mathrm{Weight}\;\mathrm{of}\;\mathrm{adjuvant}\;\mathrm{in}\;\mathrm{the}\;\mathrm{SLNs}\;\left(\mathrm{mg}\right)}{\;\mathrm{weight}\;\mathrm{of}\;\mathrm{SLNs}\;\left(\mathrm{mg}\right)}\right]\times 100$$

#### 2.4.4. In Vitro Release Study and Release Kinetics

The release pattern was carried out by the dialysis bag method. A total of 1 mL of MLT-SLN dispersion was put in dialysis bags that were immersed in 10 mL dissolution media (PBS, pH 6.8) in tightly closed conical flasks. The flasks were shaken in a thermostatically controlled water bath at 37 ± 0.2 °C, 100× *g* rpm. A total of 5 ml samples of the media were withdrawn at predetermined time intervals (zero, 0.5, 1, 2, 4, 6, 24, and 48 h). Samples were compensated with the same volume of fresh medium to keep the sinking condition. Samples were analyzed to determine MLT concentration spectrophotometrically. The percentage of adjuvant released was calculated in triplicate relative to the theoretical initial adjuvant content. Experiments were carried out in triplicate [26]. Data obtained from in vitro release studies were fitted to various release kinetic models (zero order, first order, Higuchi, Hixon Crowel, Korsmeyer Peppas) to determine the release mechanism.

#### 2.4.5. Determination of the Stability of the Prepared RA-SLNs

The stability study of the adjuvant-loaded SLN dispersion stored at 4 °C was analyzed at 1, 2, 3, and 6 months, then was compared with the zero-time data of the freshly prepared formulae. The particle size and the percent adjuvant entrapment of the nanoparticle dispersion were re-evaluated as stability parameters [27].

### 2.5. Animal Grouping and Vaccination Schedule

Sixty male Swiss albino strain mice were used in the study, four to six weeks old, weighing 20–25 g. The mice were housed in dry clean plastic cages with free access to water and food. This was under a day-light cycle of 12:12 under suitable room temperature and humidity. Food was composed of wheat, bread, and water on alternate days. All experimental studies were conducted in agreement with the Egyptian National Animal Welfare Standards and approved by the Ethics Committee of Alexandria Faculty of Medicine (Approval number: 0201455). The mice were equally divided into five groups (twelve mice in each group):

Group I Control groupEach mouse received sterile PBS.Group II MLT-SLNs groupEach mouse received 100 mg/kg/dose of MLT in a dispersion of SLNs [28].Group III TLA groupEach mouse received 20 µg/ dose of TLA in PBS [7]Group IV TLA/plain SLNs groupEach mouse received 20 µg/ dose of TLA in PBS and 130 µg lipid/dose of plain SLNs [29]Group V TLA/MLT-SLNs groupEach mouse received a suspension containing 20 µg/dose of TLA in PBS and 100 mg/kg/dose of MLT in a dispersion of SLNs.

**Figure 1:**
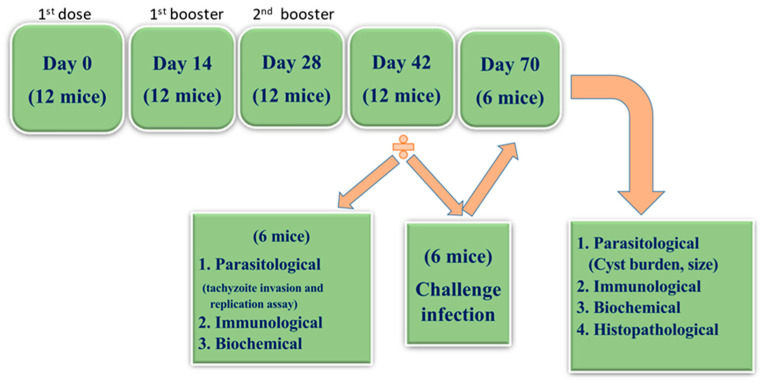
Flowchart representing the time schedule of the experimental study.

Mice of all groups were intra-nasally inoculated by being held in an upright position after being intraperitoneally anesthetized with 0.3 mL of ketamine (10 mg/mL) and xylazine (1.0 mg/mL), diluted in 0.9 % NaCl. Each mouse was given the fore-mentioned doses in a final volume of 20 µL (10 µL in each nostril) using a micropipette adapted with a tip by slowly pipetting onto the tip of the nose. The mice were kept on their backs until complete recovery from anesthesia was achieved. Each group received two booster doses with two-week intervals [7]. Two weeks following the last booster dose, six mice from each group were anesthetized with a ketamine/xylazine mixture and blood samples were drawn into Eppendorf aliquots for serum separation for subsequent studies. Thereafter, these mice were sacrificed by cervical dislocation. At sacrifice, intestinal segments and spleens were collected for subsequent immunological studies. Furthermore, brains were harvested for biochemical studies (Figure 1).

The remaining mice were infected two weeks after the second booster dose by gavage, with 100 μL of the brain homogenate containing 20 cysts of Me49 *Toxoplasma* strain (non-lethal dose) [30]. Four weeks following the challenge of the infection, mice from each group were anesthetized and sacrificed as previously mentioned. Serum, intestinal segments, brains, and spleens were collected for subsequent studies (Figure 1).

### 2.6. Vaccine Evaluation

#### 2.6.1. Parasitological Study

##### Tachyzoite Invasion and Replication Assay

This assay was performed to assess the ability of serum antibodies to mediate the inhibition of tachyzoite invasion and replication in cultured Vero cells according to the method of Wagner et al., 2015 [6]. Briefly, blood samples collected from each group on day 42 of the experiment were centrifuged at 8000× *g* for 10 min. Sera were aliquoted and stored at −20 °C. Later, the pooled serum samples collected from each group were sterile-filtered with Millex Syringe Filters (0.22 µm). Then, the sterile sera were diluted with complete culture media (RPMI1640 with L-Glutamine; 4% FCS, 1% penicillin/streptomycin) at a concentration of 2.5%. After that, tachyzoites (5 × 10^5^) were mixed with the sterile-diluted sera. Vero cells, suspended in complete culture media, were seeded in 48-well plates (4 × 10^4^ cells in 500 µL medium per well). Then, tachyzoites were added to the cultured Vero cells. After 5 days of cultivation under standard conditions (5% CO_2_, 37 °C), the medium, including free tachyzoites, was removed and the total number of tachyzoites/well was determined. A total of 10 µL of tachyzoites per well were counted in aliquots using trypan blue for live staining. The assay was carried out in triplicate.

##### Brain Cyst Count and Size

At day 70 from the beginning of the experiment, the total brain cyst burden was counted. The % reduction (%R) was determined by the following equation:

%R = 100 (C − W/C)

C: Total parasite burden recovered from the control group of mice.

W: Total parasite burden recovered from each of the remaining groups of mice.

The size of the harvested cysts from all groups was measured under the high objective lens (40×) using an ocular micrometer [31].

##### Ultrastructural Changes

After the preservation of brain cysts, harvested from three studied groups (I, III, V), in a 2.5% glutaraldehyde solution, they were examined by scanning electron microscope (SEM) for morphological changes [31].

#### 2.6.2. Immunological Study

Samples (sera, intestinal segments, and spleens) were collected from all groups at two different time intervals to assess the immunological parameters. The first interval was two weeks after the last booster immunization (day 42) and the second one was four weeks after challenging the infection with 20 *Toxoplasma* cysts (day 70).

##### Measurement of Total IgG in Serum

Serum anti-*Toxoplasma* IgG antibodies were analyzed by ELISA using mouse *Toxoplasma* antibody IgG, Tox IgG ELISA Kit (Chongqing Biospes Co., Ltd., Chongqing, China) according to manufacturer’s instructions. Optical densities (OD) were measured with a microplate reader (TECAN) at 450 nm. For descriptive purposes, anti-*Toxoplasma* IgG titers were expressed as group means ± SD of individual animal OD values, which were themselves the average of duplicate assays [7].

##### Measurement of Secretory IgA in the Intestinal Wash

The intestine from each mouse was carefully removed from the stomach–duodenum junction to the ileum-ascending-colon junction. Before collecting the samples, the mice were deprived of food for 8 h to deplete the intestinal contents [7]. The intestinal segments were washed out using 1 mL of cold PBS (pH 7.2) supplemented with 1% (*v*/*v*) anti-protease cocktail (PMSF, SIGMA). Thereafter, mucosal washes were vortexed and centrifuged (2000× *g* rpm for 30 min at 4 °C). Finally, the mucosal washes were aliquoted and frozen at −20 °C for subsequent S-IgA quantification using Mouse *Toxoplasma* antibody IgA *Toxo* IgA ELISA Kit (Chongqing Biospes Co., Ltd.) according to the manufacturer’s instructions. Optical densities (OD) were measured with a microplate reader (TECAN) at 450 nm. For descriptive purposes, anti-*Toxoplasma* Ig A titers were expressed as group means ± SD of individual group OD values, which were themselves the average of duplicate assays.

##### Measurement of the Interferon-Gamma (IFN γ) Level

The spleens from sacrificed mice of all groups were collected. Each spleen was minced in a Petri dish containing 2 mL of RPMI 1640 medium on ice and the dissected spleens were separated by sieving through a 40-μm cell strainer. The resulting single-cell suspensions were pelleted by centrifugation at 2000× *g* rpm. The supernatant was discarded and the red blood cells were lysed with a red cell lysis buffer. The containing tube was centrifuged and the pellet was washed four times using RPMI 1640 medium. Thereafter, live cells were counted in 10 µL of the suspension using trypan blue. The cells were resuspended in complete RPMI 1640 medium (supplemented with 10% fetal calf serum, 2 mM L-glutamine, 100 IU/mL of penicillin, and 100 μg/mL of streptomycin). Cell suspensions were adjusted to 2 × 10^6^ cells/mL. This suspension was divided in the wells of 96-well flat-bottomed tissue culture plates and stimulated with 20 μg/mL of TLA. The cultures were incubated for 72 h at 37 °C and 5 % CO_2_. Supernatants were collected from cultures by centrifugation at 200 rpm for 10 min and stored at −20 °C until cytokine quantification. Cytokine (INF-γ) levels were measured in the supernatants of cell cultures by capture ELISA using a mouse IFNγ ELISA kit (Chongqing Biospes Co., Ltd.,), according to the manufacturer’s instructions. OD was recorded spectrophotometrically at a wavelength of 450 nm using an ELISA reader and then the concentration of IFNγ can be calculated [7].

#### 2.6.3. Biochemical Study (Oxidative Stress Biomarker)

A total of 100 mg of the brains collected from all groups at day 42 and day 70 were processed for the determination of superoxide dismutase (SOD) and lipid peroxide (malondialdehyde, MDA). The brain samples were rinsed with a PBS solution, (pH 7.4.) containing 0.16 mg/mL heparin to remove any red blood cells. Then, the brain tissue was homogenized in 1 ml of cold buffer. The homogenate was centrifuged at 4000× *g* rpm for 15 min at 4 °C. The supernatant was collected and stored at −20°C until used. Serum samples were used for the determination of the total antioxidant capacity (TAC).

##### Superoxide Dismutase (SOD)

The colorimetric SOD assay kit (biodiagnostics, Egypt) was used to evaluate the superoxide dismutase activity in the supernatant of brain homogenates. It was measured using the method of Sun et al., 1988, according to the manufacturer’s structure. The absorbance was measured using a microplate reader at 560 nm. The values were expressed as U/mg tissue [32].

##### Malondialdehyde (MDA) (Lipid Peroxide) Assay

The amount of MDA was measured according to Draper and Hadley (1990) using the colorimetric lipid peroxidation assay kit (Biodiagnostics, Giza, Egypt) according to the manufacturer’s instruction. The absorbance was measured spectrophotometrically at 534 nm. MDA levels were expressed in nmol/mg total protein [33].

##### Total Antioxidant Capacity Assay (TAC)

Serum samples were used to measure the total antioxidant concentration using the Total Antioxidant Capacity (TAC) colorimetric assay kit. The absorbance was read at 505 (500–510 nm) using a microplate reader [34].

### 2.7. Histopathological Examination

Part of the brain tissues harvested at day 70 from all studied groups was fixed in 10 % formalin for histopathological examination using hematoxylin and eosin (H&E). Pathological changes in the brains of mice challenged with the Me49 *Toxoplasma* strain were recorded [35].

### 2.8. Statistical Analysis of the Data

Data were analyzed using IBM SPSS software package version 20.0. (Armonk, NY, USA: IBM Corp.). The Shapiro–Wilk test was used to verify the normality of distribution. Quantitative data were described using the range, mean, and standard deviation. The significance of the obtained results was judged at the 5% level. F-test (ANOVA) was used for normally distributed quantitative variables, to compare between more than two groups, and the post hoc test (Tukey) for pairwise comparisons.

## 3. Results

### 3.1. Characterization of Plain and Adjuvant-Loaded SLNs

#### 3.1.1. Colloidal Characterization (Figure 2)

**Figure 2:**
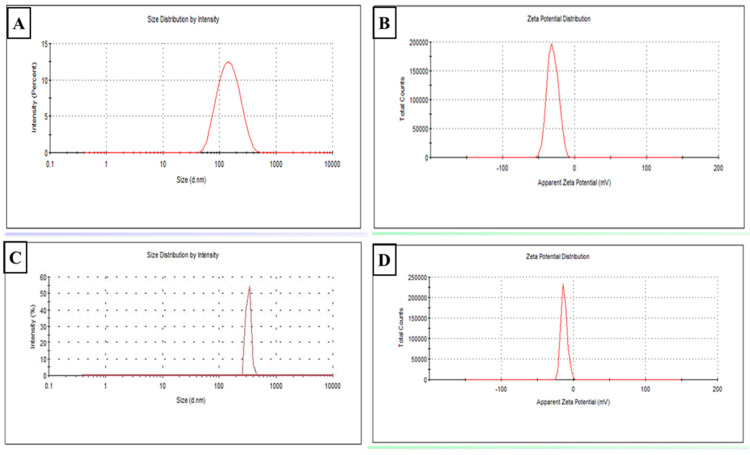
Colloidal characterization of freshly prepared plain and MLT-SLNs suspensions (at zero-day). (**A**) Particle size curve distribution for plain SLNs. (**B**) Zeta potential curve of plain SLNs. (**C**) Particle size distribution curve of MLT-SLNs. (**D**) Zeta potential curve of MLT-SLNs.

The particle size of plain SLNs was 160 ± 8 nm (Figure 2A) and the PDI was 0.16 ± 0.01 (less than 0.5, which indicates the homogeneity of the formulation). The zeta potential of plain SLNs was −24 ± 0.3 mV (Figure 2B). On the other hand, the particle size of freshly prepared MLT-SLN suspension (at zero-day) was 342 ± 7 nm (Figure 2C), the PDI was 0.23 ± 0.1 (less than 0.5), whereas the zeta potential was −20 ± 0.2 mV (Figure 2D).

#### 3.1.2. TEM (Figure 3)

**Figure 3:**
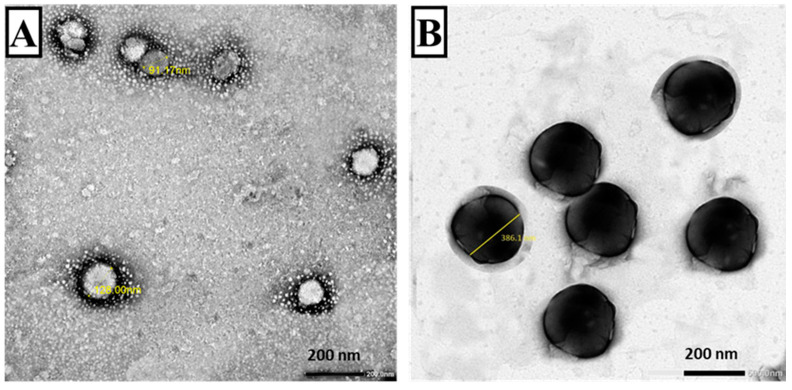
TEM of plain and adjuvant loaded SLNs (×30,000). (**A**) Plain SLNs showing rounded smooth surfaces with sizes ranging from 83.87–106.81 nm. (**B**) MLT-SLNs rounded smooth surfaces with sizes ranging from 278.12–330.4 nm.

TEM examination revealed that the freshly prepared plain SLNs were completely spherical with smooth surfaces and no agglomeration, and the diameter of the particles ranged from 91.17 to 128 nm (Figure 3A), whereas the examination of freshly prepared MLT-SLNs showed an increase in the particles size while retaining their spherical shape. The particle diameter for MLT-SLNs ranged from 298.12 to 386.1 nm (Figure 3B).

#### 3.1.3. Entrapment Efficiency and Adjuvant Loading

The freshly prepared MLT-SLNs (at zero-day) showed an EE and AL of 89.5 ± 0.3% and 19 ± 3.5%, respectively.

#### 3.1.4. In Vitro Adjuvant Release

**Figure 4:**
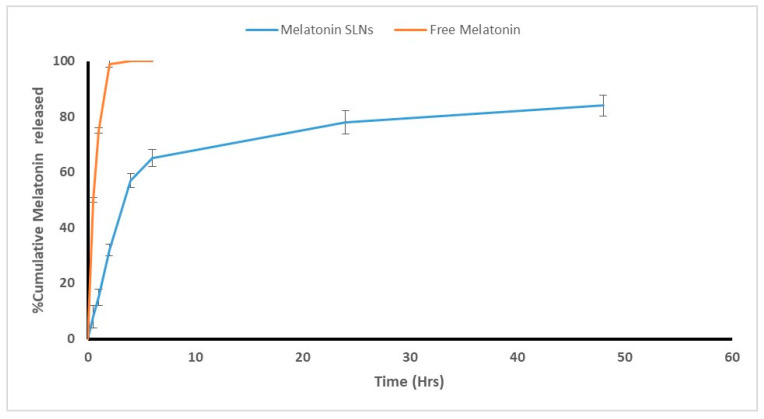
In vitro release profile of MLT from SLN dispersion versus free MLT. *n* = 3. Data represented as mean ± SD.

In vitro adjuvant release patterns were carried out using the dialysis bag method in PBS (pH, 6.8) at 37 °C. The cumulative amount of adjuvant release was plotted against time to obtain release profiles (Figure 3). The diffusion of free MLT was rapid with 99% of adjuvant released in the first two hours. On the other hand, adjuvant release from SLNs in freshly prepared suspensions (at zero-day) exhibited a biphasic pattern that was observed by an initial rapid release followed by a slower release rate. MLT demonstrated a burst release in the initial two hours, where 32% of MLT (Figure 4) was released from the outer layer of SLNs followed by adjuvant release from the lipid matrix, which was associated with a slow and controlled release, where 84% of MLT was released from SLN suspensions within 48 h (Figure 4). This resulted in a significant difference in the cumulative amount released from adjuvant-loaded SLNs and their corresponding free adjuvant (*p* ≤ 0.05).

Regarding the adjuvant release kinetics and release mechanism, MLT-SLN formulations were studied by fitting the drug release time profile with various mathematical models. The data revealed a better fit to Korsmeyer Peppas release kinetics where the r2 value for the MLT-SLNs was 0.9645 while the n value was 0.188, indicating Fickian diffusion.

#### 3.1.5. The Stability of MLT-Loaded SLNs

**Figure 5:**
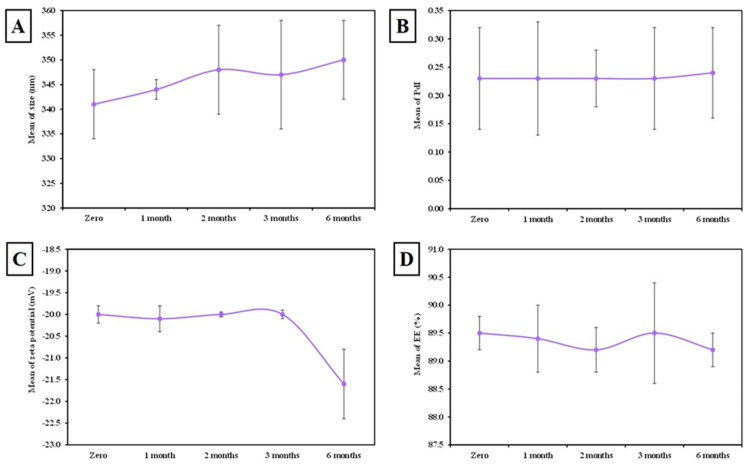
The storage stability of MLT-SLNs upon storage for six months at 4 °C using particle size analysis (**A**), PDI (**B**), zeta potential (**C**), and EE (**D**).

MLT loaded-SLN suspensions were stored at 4 °C in airtight glass vials. No evidence of precipitation or agglomeration was detected during the storage period. They remained substantially unchanged, with no statistically significant difference in their physicochemical properties (particle size, PDI, zeta potential, and EE) recorded (*p* > 0.05) (Figure 5). MLT-loaded SLN suspensions retained their colloidal stability and entrapment efficiency without adjuvant expulsions over six months of storage at 4 °C.

### 3.2. Vaccine Evaluation

#### 3.2.1. Parasitological Study

##### Tachyzoite Invasion and Replication Assay

**Figure 6:**
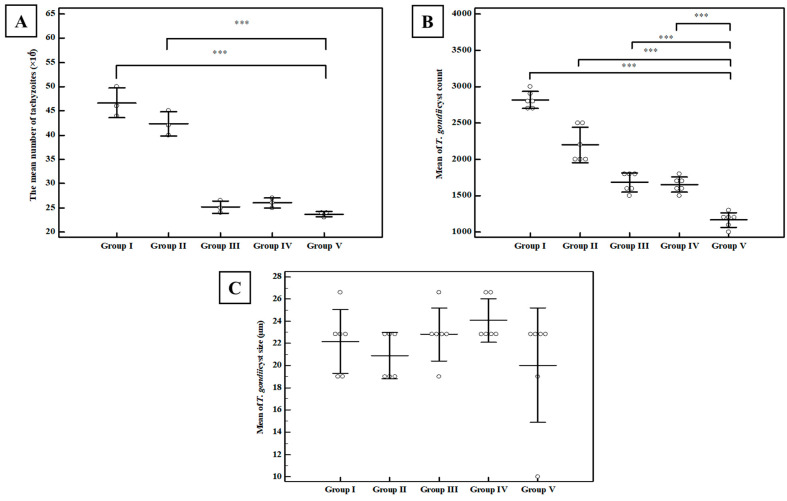
Parasitological study. (**A**) In vitro tachyzoite invasion and replication assay in Vero cells. Tachyzoites of the virulent RH strain were pre-incubated with sera from naïve mice collected on day 0, or with sera collected from mice vaccinated with different studied regimens on day 42 from the beginning of the experiment (i.e., before infection). Dots represent the mean number of *T. gondii* tachyzoites (×10^4^) per well in the culture supernatant of Vero cells after incubation. In vitro assays were performed with pooled serum samples (*n* = 3) repeated three times. Results represent data from three independent experiments, (**B**) mean of *T. gondii* cyst count and (**C**) cyst size in brain of mice of different studied groups at week 4 after the in vivo infection challenge with 20 cysts of the avirulent Me49 *Toxoplasma* strain. Data are expressed as mean ± SD. *** *p* < 0.001. Group I: Control, Group II: MLT-SLNs, Group III: TLA, Group IV: TLA/ plain SLNs, Group V: TLA/ MLT-SLNs.

Incubation of tachyzoites with sera of TLA-vaccinated mice (group III) led to a statistically significant decrease in tachyzoites count in culture supernatant as compared to the control PBS group (group I). This regimen resulted in a percentage reduction of 46.06% which was statistically significant (*p* < 0.001) (Figure 6A) (this means that only 53.9% of tachyzoites succeeded to invade Vero cells). The combination of TLA with MLT-SLNs (group V) resulted in a non-statistically significant reduction as compared to group III (TLA alone) (*p* > 0.05) (Figure 6A). 

##### Brain Cyst Count and Cyst Size

Regarding parasite burden, the mean *T. gondii* cyst count in the brain homogenate of the infected control group (group I) was 2817 ± 117 cysts. Intranasal immunization of mice with different regimens resulted in a statistically significant reduction in brain cyst count in all studied groups in comparison to the control group. Although vaccination with TLA either alone (group III) or adjuvanted with plain SLNs (group IV) showed a statistically significant reduction in mean cyst count compared to the infected control group with a percentage of reduction of 40.2 and 41.4, respectively, there was no significant difference between them. On the other hand, a marked enhancement of TLA immunogenicity with a dramatic reduction in brain cyst count was detected after the intra-nasal inoculation of TLA adjuvanted with MLT-SLNs (group V) with the highest recorded percentage of reduction of 58.6% among different studied groups (Figure 6B). Additionally, this group showed a statistically significant reduction in mean cyst count compared to either the TLA group (group III) or TLA/plain SLNs (group IV).

Regarding cyst size, the control group (group I) showed a mean cyst size of 22.17 ± 2.86 µm. Mice vaccinated with all studied regimens had no statistically significant difference in cyst size as compared to the control group (Figure 6C).

##### SEM of Toxoplasma Cyst

**Figure 7:**
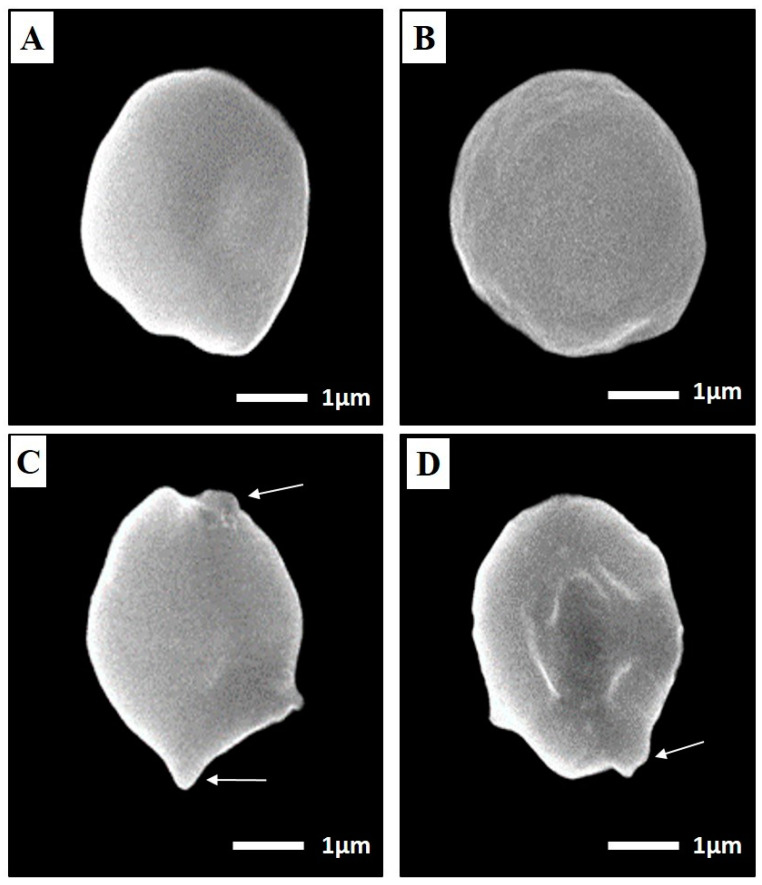
SEM of *T. gondii* cyst harvested from the infected control group (I); (**A**) mice vaccinated with TLA (group III); (**B**) mice vaccinated with TLA/MLT-SLNs (group V) (**C**,**D**). (**A**) Cysts harvested from the control group showed regular spherical-shaped bodies with some fine dimples and indentations (×12,000). (**B**) Cysts from group III showed mild roughness and surface irregularities with retained spherical outlines (×10,000). (**C**,**D**) Cysts from group V showed mild swelling and surface irregularities with a focal blebs formation (arrows) (×9500 and ×10,000, respectively).

The ultrastructural morphological features of *T. gondii* cysts in the brain homogenate of mice vaccinated with TLA alone (group III), TLA/MLT-SLNs (group V) were assessed using SEM four weeks following the challenge of the infection in comparison to the infected non-vaccinated control group (I) (Figure 7). *T. gondii* cysts harvested from the control group I appeared as spherical-shaped cysts with variable sizes and the cyst surface appeared regular with some fine indentations (Figure 7A). Meanwhile, cysts recovered from vaccinated mice with the aforementioned regimens revealed variable degrees of morphological changes. *T. gondii* cysts harvested from mice vaccinated with TLA alone (group III) showed a rough surface with fine granulations but retained the spherical outline (Figure 7B), whereas those collected from mice vaccinated with TLA adjuvanted with MLT-SLNs (group V) showed mild swelling and surface irregularities with a focal blebs formation (Figure 7C,D).

#### 3.2.2. Immunological Results

##### Total Ig G in Serum

**Figure 8:**
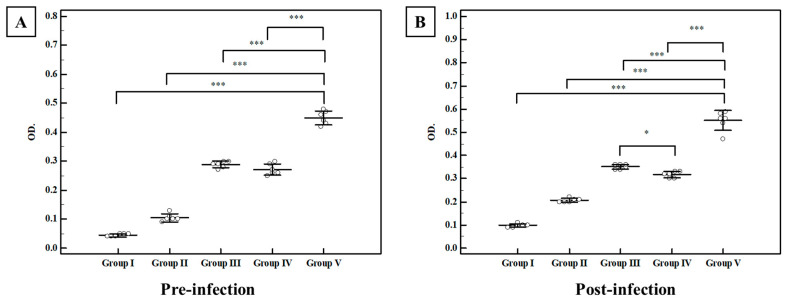
Pre-infection and post-infection serum levels of anti-*Toxoplasma* IgG (OD) among different studied groups. (**A**) Anti-*Toxoplasma* IgG antibody levels in sera of mice two weeks following the last booster dose of immunization (pre-infection). (**B**) Anti-*Toxoplasma* IgG antibody level in sera of mice four weeks after challenging the infection (post-infection). Data were expressed by using Mean ± SD. ***: Statistically significant at *p* ≤ 0.001. *: Statistically significant at *p* ≤ 0.05. Group I: Control Group II: MLT–SLNs, Group III: TLA. Group IV: TLA/ MLT plain SLNs, Group V: TLA/ MLT–SLNs.

Regarding the pre-infection levels, the control group I showed a mean level of anti-*Toxoplasma* IgG of 0.034 ± 0.007 OD. All studied regimens induced a statistically significant rise in Ig G titer compared to control group I (*p* < 0.001) (Figure 8), while the combination of TLA with plain SLNs (group IV) elicited a non-statistically significant rise in Ig G titers compared to group III (TLA alone). On the other hand, intranasal inoculation of mice with TLA adjuvanted with MLT-SLNs (group V) recorded the highest rise in anti-*Toxoplasma* IgG among all the studied groups with a mean of 0.449 ± 0.023 OD. Orally challenging the infection with avirulent Me49 *Toxoplasma* cysts promoted anti-*Toxoplasma* IgG production with retained high titers among different studied groups in a similar statistically significant manner detected at the pre-infection time (Figure 8).

##### Secretory IgA in the Intestinal Wash

**Figure 9:**
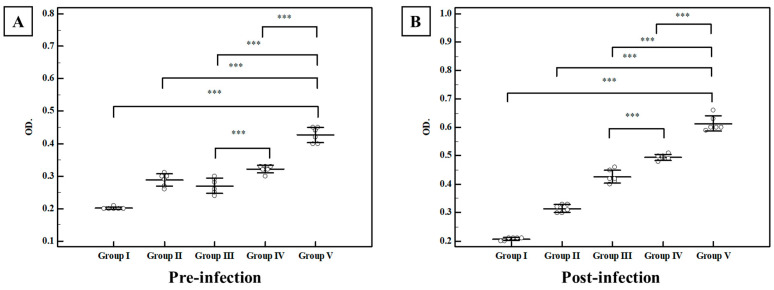
Pre-infection and post-infection intestinal levels of anti-*Toxoplasma* IgA (OD) among different studied groups. (**A**) Anti-*Toxoplasma* IgA antibody levels in intestinal washes of mice two weeks after completion of immunization schedules (pre-infection). (**B**) Anti-*Toxoplasma* IgA antibody level in intestinal washes of mice four weeks after oral challenge with Me49 *Toxoplasma* cysts (post-infection). Data were expressed by using Mean ± SD. ***: Statistically significant at *p* ≤ 0.001. Group I: Control, Group II: MLT–SLNs, Group III: TLA, Group IV: TLA/ MLT plain SLNs, Group V: TLA/ MLT–SLNs.

Intestinal washes from all studied groups have been collected and subjected to S-IgA evaluation using the ELISA assay. Regarding pre-infection levels, the mean anti-*Toxoplasma* IgA for control group I was 0.201 ± 0.004 OD. All studied regimens significantly enhanced the induction of anti-*Toxoplasma* IgA with variable degrees as compared with normal control group I. The combined regimens using TLA with plain SLNs (group IV) recorded an enhancement of mucosal immune response compared to group III (TLA alone), however, the highest mucosal antibody response with a statistically significant rise in anti-*Toxoplasma* IgA levels were detected upon adjuvanting TLA with MLT-SLNs (group V) with a mean of 0.426 ± 0.026 OD (Figure 9). Following the oral infection with *T. gondii* tissue cysts, the anti-*Toxoplasma* IgA level showed a statistically significant rise in all studied groups compared to the control-infected group I in a similar manner to the pre-infection levels (Figure 9). Again, mice vaccinated intra-nasally with TLA in combination with MLT-SLNs (group V) showed the highest rise in anti-*Toxoplasma* IgA levels among the different studied groups with a mean level of 0.612 ± 0.027 OD (Figure 9).

##### Interferon-Gamma (IFN γ) Level

**Figure 10:**
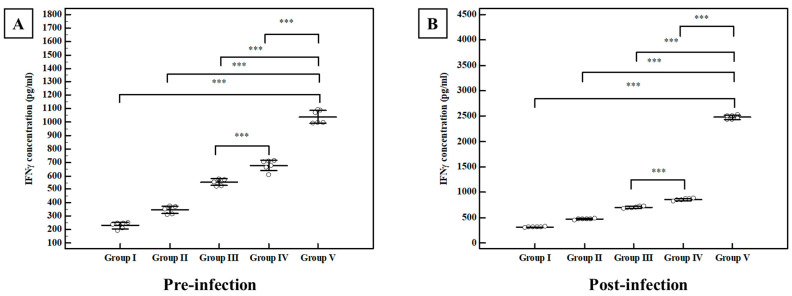
Pre-infection and post-infection splenic cell culture IFN-γ production among different studied groups. (**A**) INF-γ concentration (pg/mL) in the supernatant of splenic cell suspension two weeks after the last immunization dose (pre-infection). (**B**) INF-γ concentration (pg/mL) in the supernatant of splenic cell suspension four weeks following infection (post-infection). Data were expressed by using Mean ± SD. ***: Statistically significant at *p* ≤ 0.001. Group I: Control, Group II: MLT–SLNs, Group III: TLA, Group IV: TLA/ MLT plain SLNs, Group V: TLA/ MLT-SLNs.

Regarding the pre-infection levels, the mean concentration of IFN γ in the control group (I) was 231 ± 24.5 pg/mL. All studied regimens significantly potentiated the production of IFN γ with different levels when compared with normal control group I (Figure 10). MLT-SLNs enhanced the efficacy of TLA (group V) and showed a mean INF-γ concentration of 1038 ± 48.8 pg/mL with a statistically significant rise in the mean IFN γ concentration compared to its control groups (group I, II, III, IV). Following the challenge of the infection, all studied regimens showed an elevation in the mean IFN γ concentration compared to the infected control group I in the same manner as that reported in the pre-infection level (Figure 10).

#### 3.2.3. Biochemical Results

Two weeks following the last booster dose, brain SOD levels of the normal control group I showed a mean of 1415.33 ± 9.7 U/g tissue. No significant differences in the mean SOD levels among different studied groups compared to normal control group I were reported except in those receiving either MLT-SLNs alone (group II) or as an adjuvant in combination with TLA (group V). The antioxidant ability of MLT-SLNs as an adjuvant was evidenced by eliciting the highest significant increase in the mean SOD level which was 1722 ± 11.7 U/g tissue (Table 1). In contrast to the previous findings, challenging the infection with *T. gondii* Me49 tissue cysts triggered a significant elevation in SOD levels in all studied groups. The infected control group I showed an elevation of SOD mean level to reach 1541 ± 16.4 U/g tissue. The TLA/MLT-SLN group (group V) showed superiority among all studied groups with the highest statistically significant increase in mean SOD level to reach 2223.0 ± 27.4 U/g tissue (Table 1).

Regarding MDA levels, the pre-infection level of the normal control group (I) showed a mean MDA level of 16.92 ± 0.70 nmol/mg. None of the studied regimens achieved any statistically significant differences compared to normal control group I at this point (Table 1). Following infection, there was an increase in the mean MDA level in the brain homogenates of the infected control group (I). They showed a mean MDA level of 29.70 ± 0.59 nmol/mg. Similarly, mice vaccinated with TLA alone or combined with plain SLNs (group III, IV) also showed an increase in the mean MDA level as compared to the pre-infection level. However, the use of MLT-SLNs alone or combined with TLA (group II, V) prevented the rise in the mean MDA level with a mean MDA level of 9.73 ± 0.96 and 11.88 ± 0.26 nmol/mg, respectively (Table 1).

Concerning TAC levels, the normal control group (group I) showed a mean TAC level of 0.992 ± 0.026 mM/L at pre-infection time. The mean level of TAC significantly increased in the sera of all studied groups as compared to the control group I. The use of MLT-SLNs as adjuvants combined with TLA (group V) resulted in a statistically significant increase in the mean TAC level to reach 1.50 ± 0.01 mM/L (Table 1). Following infection, mice vaccinated with different studied regimens retained the elevated serum levels of TAC in the same statistical manner either when compared to infected control group I or compared to each other, as shown in Table 1.

### 3.3. Histopathological Results

**Figure 11:**
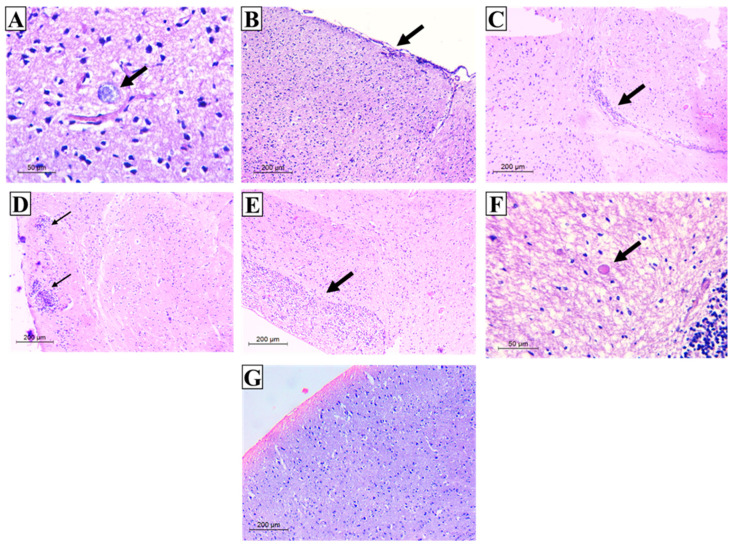
Histopathological findings in brain sections of the infected control group (**A**–**E**), and vaccinated groups (**F**,**G**). (**A**) H&E-stained section showing an oval, well-defined *T. gondii* cyst in the cerebral cortex containing multiple bradyzoites (×400), (**B**) H&E-stained section showing severe inflammatory infiltration of meninges on the surface of the cerebral cortex (×100), (**C**) H&E-stained section showing perivascular lymphocytic inflammatory infiltrate (×100), (**D**) H&E-stained section showing microglial nodules (×100), (**E**) H&E-stained section showing increased cellularity throughout the brain parenchyma (×100), (**F**) H&E-stained section showing cysts with a degenerated irregular cyst wall in the cerebral cortex (×400), (**G**) H&E-stained section showing an improvement in inflammatory changes with neither meningeal infiltration nor cerebral-increased cellularity (×100).

The H&E-stained section of the infected control group (group I) showed multiple well-defined rounded *Toxoplasma* cysts containing numerous basophilic dot-like bradyzoites (Figure 11A). Additionally, they revealed various histopathological lesions, where numerous perivascular and leptomeningeal infiltrations of inflammatory cells were observed mostly on the surface of the cerebral cortex (Figure 11B,C). This inflammatory cellular infiltrate consisted mainly of lymphocytes and few histiocytes. In addition, large microglial inflammatory nodules with increased cellularity including astrocytosis throughout cerebral parenchyma were observed (Figure 11D,E). On the other hand, variable degrees of improved inflammatory changes were noticed in the brains of mice intra-nasally inoculated with different studied regimens. Residual focal microglial nodules and mild meningeal infiltration together with fewer cysts were recorded. Furthermore, cysts appeared either with a degenerated or diffuse wall (Figure 11F). It is worth mentioning that mice vaccinated with TLA adjuvanted with MLT-SLNs (group V) recorded a marked improvement in histopathological findings where perivascular and leptomeningeal infiltrations or microglial nodules were undetectable (Figure 11G). Finally, a selective tropism of *T. gondii* toward a particular brain area following vaccination was not observed compared to the infected control group I.

## 4. Discussion

Although the human immune system is incredibly robust, it is not invincible, which is why vaccines are necessary to support the body’s defenses. The introduction of mass vaccination has saved millions of human lives and revolutionized the quality of life [36]. Nevertheless, most of the new potential vaccine candidates are characterized by poor immunogenicity, with the inability to provoke powerful and long-lasting immune responses [37]. Thus, novel adjuvants and innovative delivery systems are strongly desired to develop modern, effective, and safe vaccines [37].

Several pieces of the literature demonstrated the role of antioxidants in the management of toxoplasmosis together with their ability to ameliorate the adverse effects of vaccines [38]. Among these, melatonin (MLT) has gained exceptional attention as a natural substance with known antioxidant properties in addition to its immunomodulatory effects [39,40]. Therefore, the current work investigated the potential role of MLT-loaded SLNs as a promising mucosal adjuvant to boost TLA protective immunity together with assessing its role in modulating the pathology and oxidative stress inflicted by the infection, being a natural antioxidant and anti-inflammatory agent.

The characterization of the prepared nanoparticles revealed that the loading of MLT in SLNs resulted in a slight increase in particle size. This could be ascribed to the high lipid/adjuvant ratio (3:1) used in the preparation which was essential to enhance adjuvant encapsulation within SLNs. Previous studies reported that the size of lipid nanoparticles is highly dependent on lipid concentration, which in turn can be explained by the tendency of the lipid to coalesce at high lipid concentrations, resulting in particles of larger sizes [41,42]. It was reported that PDI values less than 0.3 are considered ideal [43]. The PDI values in the current study were within an acceptable range, suggesting nanoparticle monodispersity.

ZP revealed a negative charge of plain and MLT-SLNs which are attributed to the carboxylic groups of the lipid composition of SLNs, owing to the relatively elevated quantity of ionizable fatty acids in the matrix of SLNs (Compritol) required for sufficient coverage of the particle surface rather than the non-ionic PVA [44]. Indeed, PVA is classified as a non-ionic polymer that contains some acetate groups besides its hydroxyl ones. These acetate groups have acidic properties that allow for PVA macromolecules to gain a negative charge, which results in more negative ZPs [45]. This indicates that, in the present study, the contribution of lipids on ZP was more evident than other factors because of the high concentration of lipids in the emulsion. This goes hand in hand with Emami et al., 2015, who revealed that the type of the lipid is the most effective variable on the ZP of the nanoparticles compared to the surfactant type [42]. The EE OF MLT in SLN in the present study was higher than that reported with previous formulations, where the EE of MLT in SLNs was 89% versus 32% in lipid nanocapsules and 75% in chitosan–tripolyphosphate nanoparticles [46,47]. This could be attributed to the use of the solid lipid content of Compritol 888 ATO. This type of lipid is composed of a combination of monoglycerides, diglycerides, and triglycerides that can create an irregular or less-ordered crystalline structure (lipid matrices) [21]. The complex nature and less perfect orientation of Compritol 888 ATO make it superior in terms of adjuvant entrapment ability compared to homogenous glycerides by leaving more space for the adjuvant to be loaded, thus, allowing for a larger proportion of adjuvant entrapment [48]. Additionally, the long chain length of behenic acid in Compritol 888 ATO enhances the intermolecular entrapment of the adjuvant by inter-chain intercalation [48]. Furthermore, the increased viscosity of the medium by increasing the lipid content resulted in the faster solidification of the nanoparticles, which prevent drug diffusion to the external phase of the medium [49]. Moreover, the high percentage of MLT EE observed in this study may be due to the high concentration of PVA (4%). It was found that the increase in the concentration of surfactant results in the increased solubility of adjuvant in the lipid with a subsequent increase in its EE [50]. The in vitro release of MLT-loaded SLNs at 37 °C revealed that only 32% of MLT was released after 2 h, while the maximal cumulative release of 84% of MLT was achieved after 48 h. Since MLT are lipophilic, they can easily diffuse through the lipid core, showing sustained release. However, the order of their release is inversely proportional to the surfactant content which highlights the role of high surfactant content in SLNs, which stabilizes the SLN as well as restricts the amount of adjuvant released outside the SLN, allowing for sustained release [51]. Moreover, increasing the amount of lipid resulted in the increased viscosity of the medium, as previously mentioned, with more rigid and solidified nanoparticles that retarded the adjuvant diffusion to the dissolution medium [42]. Therefore, the MLT encapsulation is advantageous compared with its free form in terms of improved bioavailability and better in vivo half-life. The stored MLT-SLN dispersions showed neither agglomeration nor precipitation after six months of storage at 4 °C with retaining physicochemical properties (size, PDI, Z-potential, and %EE). This could be owed to the existence of a non-ionic surfactant (PVA) that boosted the stability of SLNs by decreasing the electrostatic repulsion between particles and sterically stabilizing the SLNs over the storage period [52]. This result indicated the good shelf life of the dispersions, suggesting that MLT-SLN formulations could be a promising potential adjuvant delivery system for the *Toxoplasma* vaccine.

Regarding adjuvant regimens safety, many reports confirmed that adjuvants elicit a tailor-made immune response to a specific pathogen in a dose-dependent manner [53]. In the current study, MLT was administered at a dose of 100 mg/kg. MLT was previously reported to be safe in experimental mice using regimens at the same concentration, whereas in higher doses (greater than or equal to 200 mg/kg), it caused significant body weight loss with high mortality in experimental mice [28]. Moreover, SLNs were proved to be of low or no toxicity due to the use of generally recognized as safe (GRAS) excipients [54].

Swiss albino mice were selected as experimental animal models, owing to the high susceptibility of mice to *T. gondii* strains [55] and their easy manipulation and cheap maintenance in comparison to larger animals, such as livestock and domestic animals. Moreover, the immunology of mice is well characterized. Additionally, sequencing of the mouse genome has shown that almost 99% of mouse genes have a homolog in the human genome together with a proven histological similarity of *Toxoplasma* infection in mice to that of men [56].

The intranasal administration of MLT-SLNs significantly enhanced the TLA immunogenicity in the defense against *T. gondii* infection. This enhancement could be explained by the fact that TLA, when administrated alone, is unable to protect against toxoplasmosis as the majority of non-living vaccines are relatively poor inducers of adaptive immunity unless effective adjuvants are co-administered [8,57].

Many works of literature support the involvement of antibodies in the defense against pathogens via different mechanisms, including the inhibition of adhesion to host cells [58]. Several studies showed that *Toxoplasma*-specific antibodies can inhibit tachyzoites invasion of enterocytes in vitro [59,60,61]. Results of the present work revealed that sera derived from TLA-vaccinated mice, either alone or combined with an adjuvant, reduced the invasion and replication of tachyzoites in Vero cells in vitro with variable degrees. These results are in agreement with Wagner et al., 2015, who proved that sera derived from TLA-vaccinated mice resulted in a reduction in tachyzoites invasion and replication [6].

The determination of the parasite burden by recording the brain cyst count in the brain homogenate fluid was used as a marker for successful protective vaccines against toxoplasmosis. Clearly, MLT is the main enhancer of TLA humoral immunogenicity rather than plain SLNs. It was observed that mice from the TLA/MLT-SLNs group (group V) recorded a statistically significant brain cyst count reduction compared to the TLA group (group III). On the other hand, no statistically significant difference was detected between the TLA group and TLA/ plain SLNs (groups III, and IV). Humoral, mucosal, and cell-mediated immune responses in the different groups of mice were analyzed to verify the possible underlying explanations for such superiority.

Regarding serum anti-*Toxoplasma* IgG levels, the current study proved the successful mucosal adjuvant role of the intra-nasally administered MLT in inducing strong systemic humoral IgG antibody responses. Our results go hand in hand with those of Wang et al., 2017, who reported that systemic humoral IgG antibody responses induced by the intraoral and intranasal vaccination pathway were analogous to those induced by the other pathways including parenteral ones in mouse sera [62]. On the other hand, IgG antibodies in the intravenous vaccination increased very sharply at first but slowly later on, even after the boost immunization. This could be explained by the immune tolerance resulting from the direct antigen transmission to the bloodstream that can induce strong immune responses in the early stage, however, the antibody levels increase slowly in the following time, unlike the intranasal route [63]. The failure of plain SLNs in group IV to elicit any statistically significant difference in IgG levels compared to group III (TLA alone) is in contrast with the recent results published by Shirai et al., 2020. They reported the adjuvant role of lipid nanoparticles (LNP) that could increase IgG responses with enhanced protection against influenza virus infection [29]. Those contradictory results could be attributed to the different formulations and sizes of SLNs used in the current study from the lipid nanoparticles (LNP) used in the former one together with the different types of antigens used. On the other hand, MLT-SLNs resulted in a statistically significant elevation in serum anti-*Toxoplasma* IgG levels at both pre-infection and post-infection times when administered either alone (group II) or adjuvanted to TLA (group V). This is evidence that MLT is an efficient adjuvant to B-cell epitopes when combined with the antigen of interest. However, the clinical outcome of toxoplasmosis depends on a complex balance between the host immune response and parasite virulence factors [64]. This could explain the discordance of outcomes regarding the in vitro study using the highly virulent RH strain, and the in vivo study using the avirulent Me49 strain [65]. The successful role of MLT in eliciting a strong humoral response could be attributed to the fact that MLT can increase IgG-expressing B cells by promoting the survival (anti-apoptosis) of precursor B lymphocytes in the bone marrow [18]. MLT was reported to notably increase antibody titers and IgG levels in sheep vaccinated against *Dichelobacter nodosus* [18]. Similarly, Regodón et al., 2012, reported that MLT induced an increase in serum antibody levels in vaccinations against *Clostridium perfringens* type D in sheep [66]. The anti-*Toxoplasma* IgG antibodies could control the infection through the inhibition of parasite replication and attachment to the host cell receptors. Additionally, they facilitate the attachment of the parasite to immune cells and promote its intracellular killing by macrophages via the process of antibody-dependent cell-mediated cytotoxicity (ADCC) [67].

It has been widely reported that mucosal IgA is crucial for the maintenance of mucosal homeostasis which could help in the pathogen elimination and limitation of *T. gondii* spread to other tissues, especially the muscles and brain [68]. Mucosal IgA includes antibodies that recognize antigens with high- and low-affinity binding modes, so it can neutralize inflammatory microbial products, such as enzymes and toxins, inside the epithelial cells and agglutinate microorganisms facilitating their disposal by mucociliary flow or peristalsis [69,70]. Additionally, the pathogen-specific secretory IgA antibody at mucosal surfaces is the most abundant antibody isotype and the main humoral mediator. It is considered the first line of defense against infectious agents through its ability to undergo transcytosis across epithelial cells [71,72]. Moreover, secretory IgA is involved in the protective response against *Toxoplasma* following natural infections by blocking the infection of host cells by tachyzoites and inhibiting *T. gondii* replication in enterocytes during transcytosis. Thereby, the successful induction of effective mucosal immune responses against *T. gondii* at the gut level could limit the severity of the infection [73]. The enhancement of mucosal immune response recorded in the present work goes hand in hand with El-Malky et al., 2014, who reported that the high pre-challenge levels of intestinal IgA in intra-nasally vaccinated mice can neutralize or block the orally administrated *Toxoplasma* cysts [7].

Next to antibodies, cytokines are considered another important line of parasite defense that can orchestrate the innate and adaptive host defense mechanisms during infection with *T. gondii*. Therefore, IFN-γ was used as a marker for cell-mediated immunity [6]. In the present study, MLT-SLNs enhanced the immunogenicity of TLA (group V) with the highest statistically significant rise in INF-γ concentration in splenocyte suspension culture supernatant among different studied groups. An important explanation for this effect is the intensified, specific, and vigorous responses elicited by nano-encapsulated adjuvant combined with specific vaccine antigens due to their ability to specifically target the dendritic cells (DCs), together with enhanced cellular uptake and antigen presentation [74]. Moreover, MLT was proved, by Rahimi et al., 2017, to be a promising adjuvant of DNA vaccines against human papillomavirus (HPV)-associated cancers that significantly induced strong HPV16 E7-specific CD8+ cytotoxicity and IFN-γ response [28]. The ability of the vaccine to boost a potent and broad systemic Th1 cell response that provides protective immunity against *Toxoplasma* infection was reported in several previous studies. Th1 cellular immunity depends chiefly on the ability of T cells to yield IFN-γ, which controls both acute and chronic *Toxoplasma* infection. IFN-γ is valuable in both acute and chronic *T. gondii* infection as it can restrict the growth of parasites in the acute phase and prevent its reactivation from dormant cysts in the chronic phase through IFN-γ-mediated nitric oxide (NO) synthesis in different host cells such as macrophages [75]. Moreover, IFN-γ enhances host resistance to *T. gondii* through the initiation of STAT-1-dependent expression of multiple antimicrobial genes and p47 GTPases. These GTPases belong to a class of proteins that have been widely implicated in resistance to intracellular pathogens in mice and mediate cell autonomous resistance to *T. gondii* [76]. Consequently, p47 GTPases accumulate in activated astrocytes around vacuoles containing *T. gondii* and initiate the disruption of the parasitophorous vacuole, leading to the destruction of the parasite itself [77]. It is well known that CD8+ cytotoxic T cell populations are the major effector cells against *T. gondii* being the main producers of IFN-γ [78]. Tiwari et al., 2019, revealed that CD8+ T cells can remove tissue cysts of *T. gondii* by perforin released from their lytic granules and inserted in cyst walls, leading to the formation of pores that facilitate cyst invasion with subsequent elimination. This pore-forming activity of perforin can elicit cyst wall damage, hence, morphologic deteriorations and destruction were exhibited by cysts invaded by the T cells [79].

The aforementioned immunological findings were further reinforced by the ultrastructural variations recorded in a substantial number of *Toxoplasma* cysts obtained from mice vaccinated with TLA either alone or adjuvanted with MLT-SLNs. These abnormalities could be attributed to robust cell-mediated immune responses. CD8+ cytotoxic T cells were reported to have the ability to infiltrate into a large target such as *T. gondii* cysts, where they were envisioned through the cyst wall or even fully located within the cysts in the brains of infected mice. This could help them employ their direct cyst removal effect via CD8+ cytotoxic T cell perforin-mediated cyst burden reduction along with morphological alterations [79]. It was assumed that the observation of bending the cyst wall inward is most likely due to the pressure exerted by the CD8 T cells in an attempt to penetrate the cysts [79].

A serious production of ROS with the generation of oxidative stress (OS) results from the host immune response against *T. gondii* infection. Despite the vital role of ROS against *T. gondii*, their accumulation in potentially harmful levels can elicit deadly effects inflicting tissue damage and pathology [80]. Moreover, the antioxidant/oxidant balance can intensely affect the immune cell functions. Since the brain has abundant lipid content, high energy requirements, and weak antioxidant capacity, it is an easy target for excessive oxidative insults with increased susceptibility to neuronal damage and functional decline mediated through brain oxidation [81]. Consequently, protection against oxidative damage and maintenance of adequate cellular function could be achieved by the critical inclusion of ROS inhibitors in vaccine formulations against *T. gondii* [82]. Additionally, vaccine formulations could benefit from antioxidant adjuvants by keeping low concentrations of oxidant agents to avoid the oxidation of IgG. It was observed that lower levels of oxidant biomarkers were accompanied by increased specific IgG antibodies that probably result in less damage and oxidation of immunoglobulins and even more efficient IgG production [83].

SOD is an important pillar of the antioxidant defense system, which is a key enzyme that acts as the first line of defense against ROS. Indeed, MLT has been reported to increase the stimulation of genes encoding for some antioxidant enzymes including SOD. Furthermore, its function is documented as an electron donor that directly scavenges several kinds of free radicals such as superoxide anion radical (O_2_^−^) and nitric oxide (NO). Therefore, MLT could upregulate anti-oxidative enzymes that transform free radicals into innocuous agents [84]. Following the challenge of the infection with *T. gondii* ME49 tissue cysts, an elevation in SOD level in the control infected group I was recorded. This is consistent with Türkoğlu et al., 2018, who observed elevation in SOD levels 30 days after *Toxoplasma* infection in the brain, indicating an increase in antioxidant defense mechanisms [85]. It was presumed that this compensatory antioxidant defense mechanism could even be used for diagnosis. Similarly, infection in mice immunized with different studied regimens triggered a significant elevation in SOD level compared to the control group. However, mice vaccinated with TLA adjuvanted with MLT-SLNs recorded the highest statistically significant SOD levels, indicating the role of MLT as a potential modulator of antioxidant defense hand in hand with the direct immunomodulatory effect.

The brain is highly susceptible to damage by increased OS by lipid peroxidation in which a single initiating free-radical can precipitate the destruction of adjacent molecules. This could be due to the presence of a high concentration of polyunsaturated fatty acids in membrane lipids in the brain [86]. Furthermore, antigen recognition can also be hindered by the peroxidation of membrane lipids which leads to the destruction of membrane receptors [87]. Elevation of MDA levels in brain homogenates of control group I was recorded following infection. This was expected, as many studies reported that the level of MDA is elevated in chronic acquired toxoplasmosis, which indirectly reveals the degree of oxidative cell damage [88]. Likewise, compared to the pre-infection level, mice vaccinated with TLA alone or combined with plain SLNs (group III, IV) also provoked an increase in the mean MDA level. This points to their inability to counteract the increased lipid peroxidation induced by infection. In a recent study conducted by Fatollahzadeh et al., 2021, it was perceived that mice vaccinated with TLA showed an increase in ROS levels, which might indicate its oxidant potential [89]. In the present study, the results reflected the potent antioxidant effect of MLT-SLNs in counteracting the infection-induced rise in the level of lipid peroxidation. Mice intra-nasally inoculated with either MLT-SLNs alone (group II) or combined with TLA (group V) recorded a statistically significant reduction in the mean MDA levels in their brain homogenates. Interestingly, regimens including MLT encapsulated in SLNs alone (group II) showed a better reduction. This could be ascribed to the partial decrease in MLT antioxidant effect by the TLA oxidant effect in group V. The highly lipophilic nature of MLT enables it to easily reach the cell membrane, thus, it can protect the cell membrane against lipid peroxidation. Moreover, MLT is a direct free-radical scavenger and indirect antioxidant that detoxifies a vast number of free radicals which typically increase MDA through the elevation of lipid peroxidation [90].

Correspondingly, the TLA/MLT-SLNs group (group V) recorded the highest statistically significant increase in TAC level. This was predictable, owing to its impact on the antioxidants and enzymatic activity together with its known ability to increase gene expression levels of SOD, as previously mentioned, leading to less accumulation of ROS and higher TAC levels.

The main target of an immunization program should not only be limited to protection against infection but should also consider the modulation of the pathology caused by the infectious agent. The direct immunomodulatory and antioxidant effects of the chosen adjuvant together with its indirect role through brain cyst reduction are most probably the reason behind this surprising amelioration of brain pathology. The direct immunomodulatory effect of MLT was reflected by its ability to diminish inflammatory changes, particularly the overactivation of microglia. This could be very beneficial and promising in upcoming vaccine development and design. It was observed that brains harvested from control-infected mice (group I) in this work exhibited severe inflammatory changes with many microglial nodules and astrocytosis compared to the brains of vaccinated mice. Undeniably, most CNS pathologies are often linked with abnormal microglial activation [91]. Despite the important role of activated microglia in fighting infection, being the first line of defense against pathogens that invade CNS contributing to both innate and adaptive immune response, their persistence during the chronic state is harmful to the brain [91]. Herein, the neuroprotective anti-inflammatory effects of the nano-encapsulated antioxidant adjuvant were evident. This could be attributed to the intranasal administration of MLT-SLNs that endorsed direct nose-to-brain transport via olfactory and trigeminal nerves [54]. Additionally, the evident improvement in brain inflammatory changes is mostly related to the amelioration of oxidative stress obtained by MLT which protected the cells from free-radical damage. Infection-induced oxidative stress is considered the key mechanism involved in the pathogenesis of *Toxoplasma* encephalitis (TE) and apoptosis via ROS produced by activated microglia/macrophages at uncontrolled levels [92]. It was reported that both internal and external apoptotic pathways could be induced by oxidative stress [92]. Similarly, Greenlund et al., 1995, stated that the upregulation of SOD in neurons causes a delay in apoptosis [93].

## 5. Conclusions

This work is proposed to shed light on the possible role of the natural antioxidant, MLT, as an immunomodulator to compete against toxoplasmosis. This could revitalize adjuvant design and development via its multi-target capabilities. The current study presents an attractive experimental opportunity to enhance the immunogenicity of TLA with a new adjuvant that could diminish the adverse effects accredited to the chemical composition and interactions of already existing ones, such as alum. The results discussed here provide evidence of the pivotal antioxidant role of this natural antioxidant together with its immunomodulatory mechanisms to fight against infection-induced oxidative stress. This guarantees vaccine efficacy and safety by mitigating the harmful consequences of *T. gondii* infection. Moreover, SLNs proved to be a trustworthy adjuvant delivery platform, besides the success of the intranasal route of vaccination in proffering protection against chronic toxoplasmosis. These results could open a novel perspective for the significant application of this natural antioxidant for future immunotherapies and immunoprophylaxis policies. Although animal models enable the faster evaluation of promising vaccine candidates with a low risk to humans and lower cost, many obvious species differences may limit animal models from predicting all details of how a vaccine works in humans. Therefore, careful precautions should be considered to allow for more accurate predictions of what is to be expected in clinical trials before its extrapolation to humans.

## Figures and Tables

**Table 1:** Pre-infection and post-infection mean SOD level (U/g tissues), MDA level (nmol/mg) in brain homogenates, and TAC levels (mM/L) in sera of different studied groups.

	Group I Control (*n* = 6)	Group II MLT-SLNs (*n* = 6)	Group III TLA (*n* = 6)	Group IV TLA/SLNs (*n* = 6)	Group V TLA/MLT-SLNs (*n* = 6)	^F^ *p*
SOD						
Pre-infection	1415 ± 9.7	1433 ^a^ ± 14.0	1426 ± 4.5	1429 ± 2.8	1722 ^abcd^± 11.7	<0.001 *
Post-infection	1541 ± 16.4	1780 ^a^ ± 16.7	1753 ^a^ ± 9.4	1673 ^abc^ ± 12.5	2223 ^abcd^ ± 27.4	<0.001 *
MDA						
Pre-infection	16.92 ± 0.70	17.22 ± 1.04	16.98 ± 0.55	16.97 ± 0.22	16.85 ± 0.38	0.889
Post-infection	29.70 ± 0.59	9.73 ^a^ ± 0.96	22.88 ^ab^ ± 0.81	23.0 ^ab^ ± 0.24	11.88 ^abcd^ ± 0.26	<0.001 *
TAC						
Pre-infection	0.992 ± 0.026	1.445 ^a^ ± 0.020	1.452 ^a^ ± 0.027	1.332 ^abc^ ± 0.017	1.495 ^abcd^ ± 0.010	<0.001 *
Post-infection	1.258 ± 0.022	1.565 ^a^ ± 0.022	1.518 ^ab^ ± 0.016	1.513 ^ab^ ± 0.015	1.597 ^abcd^ ± 0.012	<0.001 *

a Pairwise comparison between each 2 groups was carried out using the post hoc test (Tukey); *p*: *p* value for comparing between the different studied groups; ^a^: Statistically significant with group I; ^b^: Statistically significant with group II; ^c^: Statistically significant with group III; ^d^: Statistically significant with group IV; ^F^: for ANOVA test. *: Statistically significant at *p* ≤ 0.05.

## Data Availability

The datasets used and/or analyzed during the current study are available from the corresponding author upon reasonable request.

## Supplementary Materials

The following supporting information can be downloaded at: https://www.mdpi.com/article/10.3390/tropicalmed7120401/s1, Figure S1. Melatonin UV spectrum; Figure S2: Calibration curve of Melatonin.
