# Supplementary material for: Nano-Encapsulated Melatonin: A Promising Mucosal Adjuvant in Intranasal Immunization against Chronic Experimental T. gondii Infection
Source: Trop Med Infect Dis. 2022 Nov 27;7(12):401. doi: 10.3390/tropicalmed7120401 (PMC9785012; doi:10.3390/tropicalmed7120401)
Supplement: Supplementary file 1 [file tropicalmed-07-00401-s001.zip › tropicalmed-2019225-supplementary.pdf]

Tropical Medicine and Infectious Disease

Supplementary Materials of Nano-Encapsulated Melatonin: A Promising Mucosal Adjuvant in Intranasal Immunization against Chronic Experimental *T. gondii* Infection

Melatonin Spectrophotometric Assay

Stock solution of melatonin was prepared in ethanol (1 mg/mL). Selected volumes of stock solution were diluted in water (0.005–0.025 mg/mL). UV scanning was done using UV Spectrophotometer (Cary 60-UV-VIS, Agilent technologies, (Koyoto, Japan) from 200–400 nm. The wavelength maxima (λ max) was detected from the spectrum (Figure S1). Absorbance of each concentration was measured at λ max detected (278 nm). Measurements were done in triplicate; calibration curve was constructed with R^2 value of 0.9998 (Figure S2).

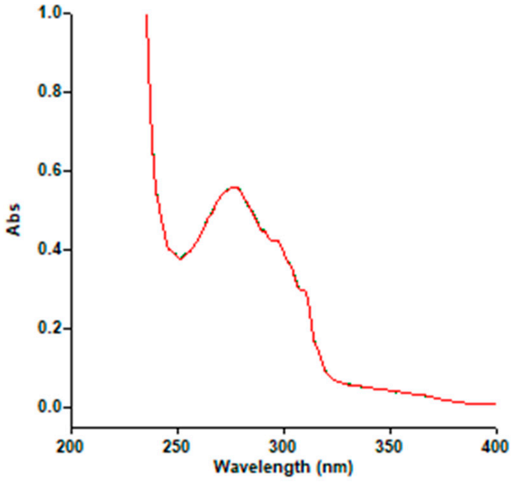

Figure S1. Melatonin UV spectrum.

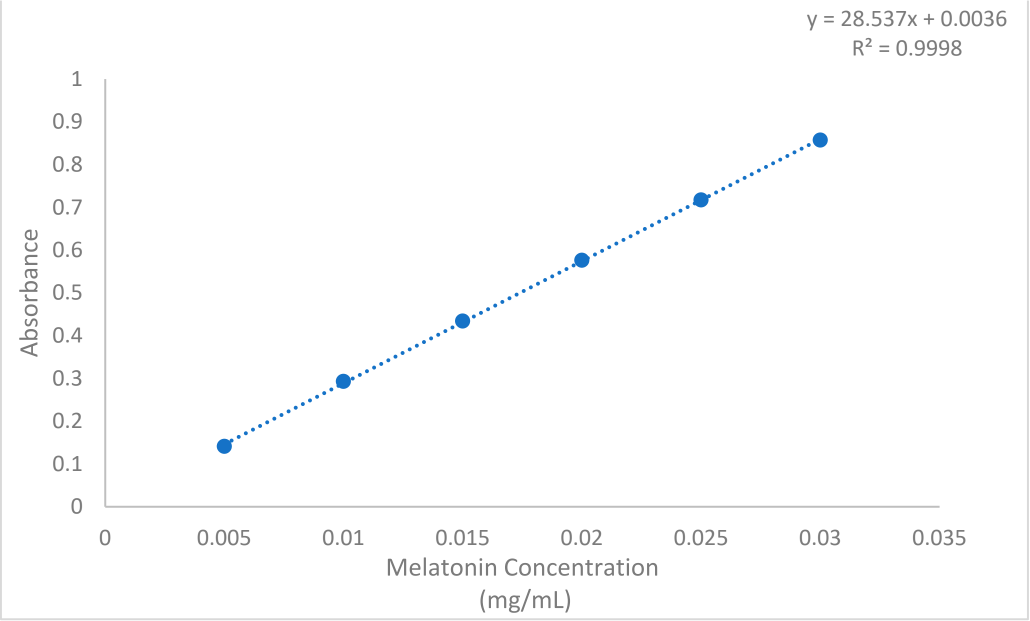

Figure S2. Calibration curve of Melatonin.
